# Congenital Pulmonary Stenosis

**Published:** 1902-09

**Authors:** Joseph Burke

**Affiliations:** Visiting Physician to the Emergency Hospital of the Sisters of Charity; attending physician to the St. Frances’ Asylum for the Aged; attending physician to the St. Vincent’s Female Orphan Asylum; consulting physician to the Widows’ and Orphans’ Asylum of the Sisters of Charity, etc., of Buffalo, N. Y., 388 Franklin Street.


### Congenital Pulmonary Stenosis.[1]

By JOSEPH BURKE, Sc. M., M. D.,

Visiting Physician to the Emergency Hospital of the Sisters of Charity; attending physician to the St. Frances' Asylum for the Aged; attending physician to the St. Vincent's Female Orphan Asylum; consulting physician to the Widows' and Orphans' Asylum of the Sisters of Charity, etc., of Buffalo, N. Y.

(*Continued from August number.*)

AFTER a perusal of the literature and with a consideration of our cases the most constant symptom of pulmonary stenosis appears to be the cyanosis. We find all grades of this cyanosis described, from the most outspoken blueness, as we find it in the cases of most severe malformation of the truncus arteriosus, to a single livid discoloration, as we find it described

1. Read before the Medical Society of the County of Erie, June 17, 1902.

in individual cases. How marked this cyanosis must be in cases of pure pulmonary stenosis cannot be absolutely determined, for there comes into consideration one circumstance above all others, and that is, that the patients first seek medical aid at a time when severe symptoms occur, where eventual adaptations, which serve to compense the mechanical disturbances, have already suffered. We must discuss the forms of cyanosis which, theoretically considered, must occur in pure pulmonary stenosis of medium or high degree, though not by atresia.

The compensatory element in pure pulmonary stenosis will lie in the hypertrophy of the right ventricle. The following phenomena and the severity of the cyanosis must depend upon the degree of this hypertrophy. By slight degree of hypertrophy or by absolute failure of such, the venous pressure must rise, the veins dilate, and we see really that in these cases secondary changes—as increase the number of erythrocytes, dilated and tortuous veins, epistaxis, hemorrhages, dilated ascending capillary network in the papillæ, richly developed capillary network in the skin in general, low temperature, clubbing of the finger ends—take place.

We might have in some cases a form of cyanosis which is due to a lack of development—a primary insufficiency—of the right ventricle. In cases of pulmonary stenosis, where the foramen ovale is open, a cyanosis can occur even by good function of the right ventricle, by hypertrophy of the same, which perhaps is partly local. A mixed cyanosis through the ductus Botalli is excluded, because the stasis is from the aorta into the pulmonary and not the reverse, as in the fetal condition. The circumstances lie differently if the right ventricle becomes insufficient. The change that results in the venous pressure must have an influence upon the cyanosis, as was formerly discussed, but this phenomenon differs from the former as well as the primary from the secondary insufficiency. Aside from the changed venous pressure, the weakness of the right ventricle must have further consequences, if, beside the pulmonary stenosis, also other disturbances of the heart, as septum defect, open foramen ovale, open ductus Botalli, are present. An existing septum defect would cause no increase of the cyanosis; the same can be said of a persisting ductus Botalli. The increase of cyanosis in these complications can result from the purely mechanical effects of the stenosis at the pulmonary orifice and the insufficiency of the right ventricle.

The conditions are different if the foramen ovale is open, for then we must expect the following: if insufficiency of the right ventricle occur then a stasis in the right auricle takes place, and as a result, this increase of blood pressure in the auricle leads to a regurgitation of the blood into the left auricle, with it to a stasis in the lungs and to an increase of the work of the already overtaxed right ventricle. This form of the *late* cyanosis is caused in a similar manner as is the cyanosis in incompensated mitral lesions. Perhaps we can explain in this manner the otherwise inexplicable dilatation of the pulmonary artery above the stenosis.

*Dyspnea.*—As the next most frequent symptom we find dyspnea. It occurs particularly in the later stages, or as a result of severe exercise. The cause of this dyspnea will always lie in the weakness of the right ventricle. The late cyanosis, already described, by open foramen ovale with pulmonary stenosis, must particularly have as a result a high degree of dyspnea. Above all, the degree of stasis in the lungs, the "Lungenstarre of v. Basch" will bring about an increase of the cyanotic character of the blood and through this an influence upon the respiratory center in the medulla. The change in the bodily warmth and the subjective feeling of coldness can doubtlessly be traced back to the cyanosis.

Also the frequency of convulsions in children with pulmonary stenosis must be mentioned as an accompanying phenomenon of this affection, and traced back to the cyanosis.

Further symptoms are various phenomena, as arrest of development and growth of the body, or especially of certain organs, particularly the genitals, so that a certain infantile habitus in many cases results. We are not in a position to decide whether there is a causal connection between the phenomena and the disturbances of the circulation which the pulmonary stenosis brings about, because we must assume in a series of cases that the pulmonary stenosis and these phenomena are an expression of a disturbance in fetal growth.

If therefore malformations in other organs occur in cases of pulmonary stenosis, it appears probable that a causal connection between both these anomalies can be thought to exist in this way: that both are the results of one cause—namely, a disturbed development. There is one factor that in some cases can play a rôle, a factor that we will later consider in detail, and one which is a frequent accompanying phenomenon of the pulmonary stenosis—namely, narrowness of the blood-vessels, particularly narrowness of the aorta.

*Kyphosis.*—Eger explained the constant presence of kyphosis in four of his cases through the weakness of the muscles of the back, which he makes responsible. Other observers also draw attention to this symptom.

It is very remarkable that hydrops occurs so seldom, particularly if one compares the pulmonary stenosis with the acquired heart lesions. The greater majority of cases die in childhood and the most of those who attain a considerable age die usually from pulmonary tuberculosis. The connection between pulmonary stenosis and pulmonary tuberculosis we will discuss later.

*Local Symptoms and Diagnosis.*—The diagnosis, intra vitam, of a congenital pulmonary stenosis in the adult has been so often made and attested to by the autopsy findings that the differential diagnosis of this lesion in classical cases causes no particular difficulty, while in the atypical cases produces often no little uncertainty, and leads to error. The constant objective signs in our three cases were as follows:

1. An outspoken rough systolic purring which could be felt most distinctly in the second intercostal space to the left of the sternum and which could be followed into the left shoulder (2) a distinct enlargement of the heart dulness to the right; (3) one could hear a rough, sawing systolic murmur over the whole heart, but the clearest in the second intercostal space to the left of the sternum; the same was transmitted in two of the cases into the left shoulder.

In reference to the behavior of the second tone it would be worth remarking that on account of the diminished blood pressure in pure uncomplicated cases of pulmonary stenosis, one should expect a weakness, or an absence, and under no circumstance an accentuation to the second pulmonary tone. Where an insufficiency exists a diastolic murmur will be audible.

4. The presence of tuberculosis in the lungs.

These four above mentioned symptoms justified us every time of making the diagnosis of a pulmonary stenosis; as also in the literature these have been the most constant signs in the typical cases.

*Diagnosis of the Complications.*—In reference to the diagnosis, whether a complication in a case of congenital pulmonary stenosis be present, there are several phenomena which point to this possibility, only the differential diagnosis of the same affords much speculation. But in spite of this, some clinicians succeed in diagnosticating the abnormal conditions that accompany the pulmonary stenosis, *e.g.*, persistence of the fetal canals, as the following will tend to show. We will first consider the presence of a septum defect.

The diagnosis in the third of our cases of septum defect made, intra vitam, and demonstrated at the autopsy, was based upon the following points: (1) On account of the age of the individual —21; (2) on account of the loudness of the first tone over the pulmonalis, an expression of the contractile power of the right ventricle; (3) on account of the intensity of the cyanosis; (4) on account of the demonstrable hypertrophy of the right ventricle.

There are cases in the literature in which, on account of the transmission of the systolic murmur into the vessels of the neck, a septum defect was diagnosticated and proved by the autopsy. In other cases the accentuation of the second pulmonary tone has been considered diagnostic. Broadbent mentions a harsh, loud, continuous murmur heard during systole and diastole, which he compares to the sound made by a knife grinder's wheel when a knife is being sharpened, and considers as diagnostic.—Broadbent, heart disease, p. 227.

*Diagnosis of Open Ductus Botalli and Foramen Ovale.*—Concerning the differential diagnosis of the persistence of the ductus Botalli or an open foramen ovale, we can consider both conditions at the same time. We must remark in the beginning, however, that in cases of pulmonary stenosis in the adult, where the stenosis is not complete, let the situation of the same be at the conus part or on the valves, the most seldom persistent fetal communication is the ductus Botalli, and on the contrary the remaining open of the foramen ovale is extremely often observed.

When we speak in the following of an accentuation of the second pulmonary sound, we wish to state emphatically that an apparent accentuation of the second sound can be caused through external agencies not lying in the heart, as thickening of the lung, retraction of the borders of the lungs, although the pulmonary sound probably shows its usual strength. When we therefore speak of an accentuation of the second pulmonary tone let it be understood that we use such a finding in the sense of a loud second pulmonary sound only.

It is further, a clinical, as well as a physiological, well-established fact, that in cases of aortic valvular stenosis the second sound is weaker than usual, or is absent, and never appears accentuated, because the blood pressure above the stenosis is less and never more than normal; but if such a stenosis is situated at the conus part then the second sound can be not only normal but even loud. The same applies to a stenosis on the pulmonary and therefore we must expect in cases of congenital pulmonary stenosis a weak or an absent, and never an accentuated second pulmonary tone, except when a conus stenosis and a complication are present. Also passive congestion in the lungs can make the sound appear louder than corresponds to the blood quantity which passes through the stenosed orifice.

Supported by this physiological basis we can consider the possibilities of the importance of an accentuated second pulmonary sound in a case of congenital pulmonary stenosis. For the existence of such an accentuation of the second pulmonary sound—if it be due to the closing of the pulmonary semi-lunar valves—three absolutely necessary factors must work in harmony: (1) The situation of the stenosis must be confined to the conus part; (2) an increase of blood pressure must exist in the pulmonary artery; (3) the pulmonary valves must be healthy and totally or partially able to close.

When, therefore, the accentuated second sound in a case of congenital pulmonary stenosis is due to the closure of the pulmonary valves, such a loudness can originate through one certain condition—namely, through an increase in the blood pressure from the left heart above the stenosis, or through a persistent ductus Botalli. This teaching that the accentuation of the second pulmonary sound, in a case of congenital pulmonary stenosis, indicates a conus stenosis and a remaining open of the ductus Botalli, although only a result of theoretical physiological discussions and not founded upon physiological facts, is so generally recognised and willingly accepted that every assertion to the contrary must be supported by the most fundamental and most convincing proofs.

We are necessitated to deviate from this so generally accepted teaching and feel ourselves justified in assuming that the presence of an accentuated second pulmonary in pulmonary stenosis can be interpreted as a constant and pathognomonic sign for the presence of an open foramen ovale.

In the discussion of this question of the significance of the second pulmonary sound, we will analyze and tabulate the accessible published cases of congenital pulmonary stenosis where the attention of the clinician has been directed to the consideration of the second tone; we will find out in how many of these cases with accentuated pulmonary tone the ductus Botalli persisted and functionated, and at the same time in how many the foramen ovale was open.

TABLE I.

| Name of Author. | Character of the Second Pulmonary Sound. | Foramen Ovale. | Ductus Botalli. |
|---|---|---|---|
| Kisel | Normal. | Open. | Closed. |
| Krönig | Not accentuated. | | Open. |
| Variot | Not mentioned. | Closed. | Open. |
| Bury | So remarkably accentuated that open ductus Botalli was diagnosed. The pulmonary valves were so intimately grown together, that they were not in condition to have caused a loud sound; the 2 aortic tone was less loud than the 2 pulmonary. | Wide open. | Closed. |
| Cassel | Loud, clapping. | Open. | Obliterated. |
| Croker, No. 1 | Split. | Open. | Open. |
| Dittrich | Short, accentuated. Valves five times thicker than normal; conus stenosed, high degree; valves insufficient. | Open. | Closed. |
| Kaulich | Well limited, distinct. | Open. | Closed. |
| Leo, No. 2 | Clear. | Open. | Closed. |
| Grummach | Remarkably loud, accentuated. | Wide Open. | Closed. |
| Wallach | Long, dull. | Wide open. | Closed |
| Weiss | Increased. | Open. | Closed. |
| Feriol | Not mentioned. | | Open. |
| Fraenkel | Not mentioned. | | Open. |
| Young | Not accentuated; distinct. | Open. | Closed. |
| Nixon | Accentuated. | Open. | Closed. |
| Vèlon | Not mentioned. | | Open. |
| Ackerman | Clear. | Open. | Open. |
| Jacobowitsch | Not clapping. | Open. | Open. |
| Bozannis | Clear. | Open. | Open. |
| Brauner | Increased. | Open. | |
| Achilles | Accentuated. Valves so intimately grown together could not have caused tone. | Not mentioned. | Closed. |
| v. Rad | Accentuated. | Open. | Closed. |
| Burke, 1 | Accentuated. | Open. | Closed. |
| " 2 | " | " | " |
| " 3 | " | " | " |

TABLE II.

| | Number of Cases. | Character of Second Pulmonary sound. |
|---|---|---|
| Ductus Botalli open—alone . . . . . . . . . . | 3 | No accentuation. |
| Foramen ovale open--alone . . . . . . . . | 6 | Accentuated in all. |
| Foramen ovale with ductus Botalli . . . . . | 5 | Accentuated in all. |
| In our cases was the foramen ovale open in . . | 2 | Accentuated in both. |
| Foramen ovale with defect in septum . . . . | 1 | Accentuated. |
| Open foramen ovale--alone . . . . . . . | 8 | Accentuated in 8. |
| Open foramen D. Botalli . . . . . . . . . . | 5 | Accentuated in 5 |
| Open ductus Botalli alone . . . . . . . . . . | 3 | Accentuated in 0. |

An analysis of these tables brings forth the interesting fact that in fourteen cases of pulmonary stenosis with certain accentuation of the second sound the open ductus Botalli was never found present; on the contrary in three cases, where the ductus Botalli was the only complication, the second tone appeared in two of them weak, and once absent. One finds in the predominance of cases, where clinically an accentuation of the second pulmonary tone was observed, an open foramen ovale, which could give rise to the idea that a certain causal connection might exist between this accentuation of the second pulmonary sound and an open foramen ovale. From a perusal of the literature in this respect, we can see some further interesting factors, particularly this one, that the origin of a second pulmonary sound as a valvular tone, judging from the post-mortem finding, is impossible, as various authors emphatically testify. We also must mention that in our three cases the conditions were similar. For, in the first of our cases, the valves were so changed from the endocarditis that the cusps were grown together and so stiff that not only a high degree of stenosis at the conus was present, but also a stenosis and insufficiency of the valves as well; according to the anatomical findings it must have been impossible for these valves to have produced a tone; at any rate, an accentuated one. In the third case there was a high degree of conus stenosis, "for the first finger penetrable, with apparently two cusps, one of which was delicate and slightly thickened on the free border, while on the other a hard, and irregular thickening appeared on the insertion of the cusp from the sinus Valsalva;" therefore it was hardly possible that the second sound, which appeared so accentuated, was caused by the closing of these valves. Apart from this there was not a sign of a persistent ductus Botalli. In our second case, I am sorry that a detailed description in the post mortem records is wanting, therefore it would be hardly possible to judge whether the valves could produce a sound or not. At any rate, they were stenosed and insufficient, and could hardly have given rise to an accentuated tone.

In many of the above cited cases in the literature, *e.g.*, in the case of Bury, where the second sound was so remarkably accentuated that that observer diagnosticated a persistent ductus Botalli, the valves were so adherent that they could not have given rise to a tone; and in Dittrich's case, where the second tone was short and accentuated, the valves were five times thicker than normally and insufficient, the conus highly stenosed, all of which evidence tends to prove that the pulmonary valves could not have caused the accentuation heard in these cases. If the accentuation of the second sound was not due to the closing of the pulmonary semilunar valves and the persistence of the ductus Botalli, then there exist three other possibilities to explain its origin: (1) It could have been transmitted from the aorta; (2) it could be caused by the contraction of the walls of the pulmonary artery; (3) or by contraction of the hypertrophied auricular walls.

The fact that one would expect an increase in the arterial blood-pressure by such a transmission and ought to hear this sound with greater, or even at least equal, intensity over the aorta, would speak strongly against the first possibility; in none of the cases quoted here has this been observed; in our cases certainly it was not the fact.

There appears absolutely no positive proof for the existence of the second possibility, while for the third possibility of the mode of origin there are several signs that support such an assumption, because there is in a normal heart an auricular sound, which, on account of external conditions, is not heard, and which under pathological conditions, as in increase of blood pressure in the auricle, becomes audible. Besides, we find here a circumstance which, as the autopsy results prove, lead to hypertrophy of both auricles, and this is the remaining open of the foramen ovale. It might be possible therefore, that the very loud sound is produced by the contraction of the abnormally filled hypertrophied auricles.

In pulmonary stenosis with open foramen ovale an increased function of the right as well as the left auricle exists, in order to overcome the opposing pressures, to prevent an overstreaming of the blood. This fact is supported not only by the hypertrophy demonstrated at the autopsy, but also by the fact that the valvula Eustachii is so often found developed, which shows the existence of a function of the same during life--namely, the transmission of blood to the foramen ovale. If the blood which flows along the Eustachian valve meets a lower pressure in the left auricle, the venous blood must flow into the left auricle; in compensated cases, however, where the auricles are sufficiently hypertrophic to prevent this mixing of the blood, this does not take place, because the constant working of one positive pressure against another of the same intensity causes a hypertrophy, and so the contraction of these hypertrophied walls can produce a tone which is heard in the diastole or presystole according to the time of the auricular contraction. I believe, therefore, that the accentuated second sound in pulmonary stenosis, where no ductus Botalli but an open foramen ovale is present, is an auricular tone, and that the presence of an accentuated second pulmonary sound in such a case of congenital pulmonary stenosis is essentially characteristic of an open foramen ovale, but under no consideration pathognomonic of ductus Botalli.

We have in the foregoing accepted an opinion which is also shared by most authors, that in open ductus Botalli, through streaming over of blood from the aorta into the pulmonalis, the filling of the veins, as well as the pressure is raised, so that an accentuation of the second sound must occur.

In opposition to the negative finding of an increase of the second pulmonary sound as given by the statistics, there comes one factor above all others into question, which is little considered—namely, whether the lumen of the ductus Botalli in such cases permits of a sufficiently copious streaming over from the aorta into the pulmonalis. In a series of cases where it says, for instance, that the ductus Botalli would permit of a probe passing through it, such an influence of the communication must certainly be denied. If we expect that an increase in the tension in the pulmonalis, and as a result of it an accentuation of the second pulmonary tone, so must a great quantity of blood pass through the ductus Botalli from the aorta into the pulmonalis.

The origin of still another phenomenon mentioned by other observers, which occurred in two of our cases, and which can be interpreted in support of the diagnosis of an open foramen ovale—namely, the presystolic murmur, could be explained by the formation of eddies, which are caused by the coming together of the two blood currents in the auricles, especially in the end stages.

### Table III.

| No. | Name of Author. | Tuberculosis. | Character of the Aorta. |
|---|---|---|---|
| 1. | Rickards | Present | Very narrow. |
| 2. | Variot | Absent | Wide. |
| 3. | Voss | Absent | Dilated. |
| 4. | Howship | Absent | Wide. |
| 5. | Farrel-Watson | Absent | Wide. |
| 6. | Raule-Chasmal | Absent | Dilated. |
| 7. | Mansfield | Absent | Wide. |
| 8. | Mallach | Absent | Normal. |
| 9. | Dubnell | Absent | Wide. |
| 10. | Sibbold | Absent | Dilated. |
| 11. | Mannkopf | Present | Very narrow, |
| 12. | Frankel | Present | Normal. |
| 13. | Wyss | Absent | Wide. |
| 14. | Ashby | Absent | Normal. |
| 15. | Maulard-Martin | Present | Narrow. |
| 16. | Vereol | Present | Narrow. |
| 17. | Young | Absent | Narrow. |
| 18. | Casey | Absent | Normal. |
| 19. | O'Sullivan | Absent | Normal. |
| 20. | Velon | Absent | Normal. |
| 21. | Hall | Absent | Wide. |
| 22. | Krönig | Absent | Wide. |
| 23. | Nasarow | Present | Narrow. |
| 24. | Passaw | Absent | Wide. |
| 25. | Hadden | Present | Narrow. |
| 26. | Orth, No. 1 | Miliary tuberculosis | Narrow. |
| 27. | Orth, No. 2 | Present | Narrow. |
| 28. | Ackerman | Absent | Abnormally wide. |
| 29. | Jakabow | Absent | Abnormally wide. |
| 30. | Bozanis | Absent | Narrow. |
| 31. | Schmidt-Kirsh | Absent | Wide. |
| 32. | Aberle | Absent | Wide. |
| 33. | Rokitansky | Absent | Wide. |
| 34. | Schautz | Present | Very Narrow. |
| 35. | Bury | Absent | Narrow. |
| 36. | Etlinger | Absent | Wide. |
| 37. | Kaulich | Absent | Wide. |
| 38. | Kruger | Absent | Wide. |
| 39. | Leo. No. 1 | Absent | Wide. |
| 40. | Leo, No. 2 | Absent | Wide. |
| 41. | Grummach | Present | Narrow. |
| 42. | Vitte | Absent | Wide. |
| 43. | Bury | Absent | Wide. |
| 44. | Weiss | Absent | Wide. |
| 45. | Burke, No. 1 | Present | Narrow. |
| 46. | Burke, No. 2 | Present | Narrow. |
| 47. | Burke, No. 3 | Present | Narrow. |

### Table IV.

| | | | |
|---|---|---|---|
| Narrowness of the Aorta | 13 | Tuberculosis: | |
| | 3 | Present | 13 |
| | —— | Absent | 3 |
| Cases | 16 | | —— |
| Wideness of the aorta | 31 | Cases | 16 |
| | —— | | |
| Total cases | 47 | | |

As the last point of our discussion let us consider the influence which the pulmonary stenosis has upon pathological conditions in the lungs; and there we find advanced by the most prominent authors that congenital pulmonary stenosis leads to tuberculosis of the lungs. It is here pointed out above all others the anemia of the lungs due to the pulmonary stenosis, which is said to act as the causative factor in the origin of the tuberculosis. In opposition to this assertion there exist doubts even from a purely theoretical standpoint. For, in the first place, the lung tissue is not supplied by the pulmonary artery, but by the bronchial arteries which transmit the necessary blood quantity for the nourishment of the lungs; secondly, we see pulmonary tuberculosis comparatively seldom in acquired pulmonary stenosis. There does not appear, therefore to be a direct dependence of the phthisis on the pulmonary stenosis.

If we have recourse to the statistics and see whether in those cases of pulmonary stenosis where there could be demonstrated no change in the aorta, particularly no narrowness of the aorta, the pulmonary tuberculosis occurred so frequently, we discover from the literature that (see accompanying Tables III. and IV.) in 48 reported cases, with mention of the aortic circumference, 16 cases had narrowness of the vessels, particularly the aorta, in 13 of which pulmonary tuberculosis was present; in contrast to this, in 32 cases where wide, or normal volume of the aorta was present, no trace of tuberculosis was demonstrable.

We have already mentioned above that we are obliged to explain other malformations which we find in the majority of cases of pulmonary stenosis as coördinate phenomena of a disturbance of the fetal development, and it does not appear wonderful therefore that we see occurring in a series of cases where this development was arrested phenomena which likewise are only results, as the pulmonary stenosis itself, of this developmental disturbance. Such is according to our opinion the tuberculosis of the lungs in people who have pulmonary stenosis.

The tuberculosis is due to a developmental anomaly—namely, congenital narrowness of the aortic system.

In conclusion, we would like to recapitulate briefly the logical results of our discussion.

We feel ourselves justified in assuming that:

1. The theory of Kussmaul, "Die Correctur der Stauungstheorie," appears to us the most probable, as the etiological factor for the origin of the pulmonary stenosis, because it sees a cause in the embryological arrest in development, as well as in the fetal inflammatory phenomena, for the development of a congenital pulmonary stenosis.

2. Through the pulmonary stenosis itself, a series of malformations of the heart in the fetal condition, can be promoted, as must be critically inferred.

The pulmonary stenosis of moderate degree can promote the remaining open of the foramen ovale, as well as eventually the persistence of a septum defect. The ductus Botalli remains uninfluenced or even hindered, by the pure physical narrowing of the pulmonary stenosis. The statistics agree with this assertion and demonstrate that the foramen ovale occurs very frequently, the ductus Botalli quite seldom in combination with congenital pulmonary stenosis.

In the extra fetal state the pulmonary stenosis affects the right ventricle. Where there is a lack of development of the same we see a condition occur which corresponds to a primary insufficiency. The result of this is stasis in the veins, cyanosis, epistaxis, dilated venous network, and the like.

By good tendency of the same so that a compensatory hypertrophy can occur, the pulmonary stenosis can cause a mixed cyanosis, particularly in open foramen ovale.

Whether a similar occurrence, a mixed cyanosis, can take place by septum defect, we will leave the question unsettled, *sub judice.* This circumstance changes immediately if an insufficiency of the right ventricle occurs. In this case the so-called late cyanosis can occur through this secondary insufficiency. The same can be due, first, to the increase of the venous pressure, of the venous stasis, of the slowing of the venous current; second, there exists in open foramen ovale a further possibility of the occurrence of the increase of the cyanosis and dyspnea through the following circumstance: as a result of the weakness of the right ventricle, a damming back into the right auricle takes place; from there the blood passes through the open foramen ovale into the left auricle, a stasis in the lungs results, which through the increased blood pressure in the lung capillaries causes a further claim upon the injury of the right ventricle. This condition of the lung—Lungenstarre—as well as stasis in the general venous system, must have cyanosis and dyspnea as results, as we find them described by a number of authors in literature.

3. Upon the basis of our analysis of the literature and of our own cases, we conclude that the assertion made by authors that in pulmonary conus stenosis, an accentuation of the second pulmonary sound proves the existence of a coëxisting open ductus Botalli, although possible, yet appears supported by absolutely no facts. On the contrary, we find in the literature in two cases of conus stenosis with open ductus Botalli, no accentuation of the second pulmonary sound. Against this assertion we believe that we may assume an open foramen ovale as sufficient reason to explain the accentuation of the second pulmonary sound. In support of this there attest eight cases of pulmonary stenosis where there was an isolated occurrence of the open foramen ovale, the second sound was accentuated.

There are cases in the literature as well as two of our own which tend to prove that the accentuated second pulmonary sound is not due to a closing of the pulmonary semilunar valves. It might be possible that in these cases a tone of other origin, *e. g.*, an auricular sound could be mistaken for a second pulmonary tone. We must therefore refuse to accept the assertion that an accentuated second pulmonary tone in congenital pulmonary stenosis proves the existence of an open ductus Botalli, and affirm that such an accentuation is diagnostic of an open foramen ovale.

4. The statistics of the cases of pulmonary stenosis show that we can demonstrate a narrowness of the aorta also with the pulmonary stenosis, in a series of cases. This coördinate phenomenon of the pulmonary stenosis, probably due to an arrest in intrafetal growth appears, as far as the statistics do not lie, to be a particular cause for the frequent occurrence of the complicating pulmonary tuberculosis. This fact would agree better with the postulated anemia of the lungs—because such a condition could be caused by a narrowing of the vessels—as well as with the assertion of Benecke, who pointed out in an especial manner the frequency of the congenital narrowness of the vessels in tuberculosis.

### LITERATURE.

Morgagni, J. B. De sedibus et causis morborum, etc. 1761.

Sandifort. 1777. England.

Hunter, John. Three cases of malformation of the heart, etc. Transactions of Physicians in London, 1784. Vol. 6, p. 305.

Meckel, Johann. De cordis conditionibus abnormibus. 1802.

Peacock. On some of the causes and effects of valvular diseases of the heart. Cronian Lectures, London, 1865.

Lysine, R. Large communication interauriculaire et perforation de la cloison interventriculaire, absence de cyanose; retrecissement de l'Art. pulmonal. tuberculose.
Mannkopf, E. Ueber Stenose des Ostium arteriosum der rechten Herzkammer. Annalen des Charité Krankenhauses, 1862.
Moritz, F. Ueber Stenose des Pulmonalostiums München. medic. Wochensch., 1892, p. 594.
Moulard-Martin, R. Retrecissement de l'Art. pulmonal., comm. interventriculaire, tuberculsation pulmonaire. L'Union Medicale, 1883, p. 825.
O'Sullivan, S. Report of a case of congenital malformation of the heart, stenosis of the conus arteriosus with an opening between the ventricles, the foramen ovale unclosed. Journal of Medical Science, 1880, p. 350.
Rickard, E. Stenosis of the pulmonary valve, etc. The British Medical Journal, Vol. 1, 1881.
Variot et Gompert. Cyanose avec malformation congenitale du coeur, etc. Gazette des Hospitaux, LXIII.
Voss. Cyanosis congenita. Norsk Mag. f. Lar. 1856. I., p. 670.
Howship. Meckel's Archiv., I. 1815, p. 233.
Farel et Watson. Meckel's Archiv. 1815.
Raoul. Arch. Gen. Med., II. May, 1836.
Mansfield, Hamburg. Zeitschrift f. d. gesammte Heilkunde, Bd. 23. Heft 2.
Schmiedt. Ueber angeborene Pulmonalstenose. Bonner Dissertation. 1892.
Schautz, E. P. Ch. Ein Fall von Atresie der Art. pulmonalis. Marburger Dissertation. 1880.
Kaulich, Jos. Zur Diagnose der angeborenen Herzfehler. Prager med. Wochenschrift, 1884. IX., p. 505.
Krüger, G. Ein Fall von Stenosis arterial pulmonalis. Correspondenzblatt für schweizer Aerzte, 1884. XIV., p. 177.
Leo, Hans. Ueber einen Fall von Entwicklungshemmung des Herzens. Virchow's Archiv., 1886. Bd. 103.
Weiss, Sal. Ueber einen Fall von angeborener Stenose der Pulmonalis. Deutsch. Archiv. f. klinisch. Medicin, 1875. XVI., p. 379.
Holl M. Beitrag zu den Defecten des Septum ventricularum cordis. Wiener medic. Jahrbuch, 1880, p. 453.
Nasarow D. Ein Fall eines angeborenen Herzfehlers, etc. Ref. Centralblatt f. innere Med., 1897, p. 14.
Hodden. Transactions of Pathological Society. Dublin. 1861. P. 91.
Orth. Zwei Fälle von Defect im Septum ventricularum neben Verengerung des Lungenarterienostiums. Virchow's Archiv., 1880. Bd. LXXXII., p. 529.
Kirsch. Ein Fall von congenitaler Pulmonalstenose. Bonner Dissertation, 1889.
Passow. Ein Fall von Stenose des Conus arter. dexter mit Defect im Septum ventriculorum. Charité Annalen, 1894, p. 219.
Sandby, R. A case of pulmonary stenosis with patent foramen ovale. British Medical Journal, Vol. 2, 1897, p. 378.
Kisel, A. Angeborene Herzdefecte bei Kindern. Ref. Jahrbuch f. Kinderheilkunde, etc., 1888, XXVII., p. 220.
Cassel. Berlin. klin. Wochenschrift, 1891, p. 1221.
Coocker, R. Three cases of congenital malformations of heart. Medical Times and Gazette, 1879, Vol. 1, p. 189.
Weiss. Ueber einen Fall von angeborener Enge der Pulmonalis. Deutsch. Archiv. f. klin. Medicin, 1875, XVI., p. 379.
Krönig. Ein Fall von Stenose des Conus arteriæ pulmonalis. Berlin. klin. Wochensch., 1887, p. 961.
Feriol. Stenose pulmonaire avec communication des deuse ventricules cyanose tardive et intermit. tent, tuberculsation pulmonaire intime. L'Union Medicale, 1881, p. 361.
Frenkel B. Cyanose congenitale, retrecissement de l'art pulm. persistance du tr—— d. Botalls tuberculose pulmonaire. Bulletins d. l. sociètè anatomique de Paris. 1896, p. 306.
Grummach, E. Ueber angeborene Dexioeardic mit Pulmonalstenose und Septumdefect des Herzens ohne situs transversus. Berlin. klin. Wochenschrift, 1890, p. 22.
v. Basch, S. Physiologie und Pathologie des Kreislaufes, Wien. 1892.
Eger. Bemerkungen zur Pathologie und Pathogeness der angeborenen Herzfehler. Archiv. f. Kinderheilkunde, 1891. XII,, p. 348.
Dittrich, F. Die wahre Herzstenose erläutert durch einen Krankheitsfall. Prag. Vierteljahrschrift f. praktische Heilkunde, 1849. VI., Bd. I. S. 157.
Meyer, H. Virchow's Archiv. Bd. XII. Heft VI. Berlin, 1857.
Kussmaul. Ueber angeborene Enge und Verschluss der Lungenarterienbahn. Zeitschrift f. rationelle Medic., 1866. Bd. XXVI., p. 99.

Rokitansky, C. v. Die Defecte der Scheidewände des Herzens, Wien, 1875.
Brauner G. Ein Fall von Septum Defect und Stenose der Pulmonalarterie. München. Dissertation. 1892.
Bury, J. S. Congenital constriction of orifice of art. pulm. from fusion of the valves, foramen ovale open. The Lancet, Vol. II., 1884, p. 183.
Busy, S. Cyanosis, congenital abnormality of the heart. Two cases; one autopsy. American Journal of the Medical Sciences, 1880. LXXIX., p. 159.
Collier, W. Malformation of the pulmonary valves (simulating aneurism of the aorta): The Lancet, Vol. 1, 1888, p. 981.
Achilles. Ein Fall von Pulmonalstenose. Würzburg. Dissertation, 1879.
Ackermann, A. Ueber congenitale Pulmonalstenose. Hallenser Dissertation, 1869.
Ashby, H. Congenital heart disease; atresia of the P. A.; patent foramen ovale and duct. art. The Medical Times and Gazette, Vol. 1, 1884, p. 353.
Bozannis, G. D. Ein Fall angeborener Pulmonalstenose. Würzburger Dissertation, 1876.
Eger. Bemerkungen zur Pathologie und Pathogenese der angeborenen Herzfehler. Deutsche medic. Wochensch., 1893, p. 81.
v. Etlinger. Zur Casnistik der angeborenen Herzfehler. Archiv f. Kinderheilkunde, 1891, XII. p. 348.
Jadubowitsch, Anna. Ein Fall von Atresie der Art. Pulm. Züricher Dissertation, 1897.
Young, Josephine. A case of defect in the ventricular septum et stenosis of the pulmonary conus in a man 32 years old. Medicine, 1897, for June.
Wallach. Archiv für Physiologie, XI., 1852.
Dubrucil. Paris, 1847, p. 22.
Wyss, O. Ein Fall von stenosis arteriæ pulmonalis. Correspondenzblatt f. schweizer Aerzte, 1871, I., p. 43.
Cassy. Cyanose congenitale, etc. Le Progrès médical, 1878, p. 263.
Vilon. Le Progrès médical, 1885, p. 423.
Nixon, J. Post-mortem appearances found in a case of cyanosis. The Dublin Journal of the Medical Science, 1879, Vol. LXVIII., p, 417.
Kisel, A. Ejenedielnaja klinicheskaja Gazeta, 1887, VII., p. 146.
Krönig. Ein Fall von Stenose des Conus arteriæ pulmonalis. Berliner klinische Wochenschrift, 1887, p. 961.
Variot. Gazette des Hopitaux, LXIII., p. 315.
Bury. The Lancet, Vol. 2 for 1884, p. 183.
Cassel. Berliner klinische Wochenschrift, 1891, p. 1221.
Croker.. Medical Times and Gazette, Vol. 1, 1879, p. 189.
Dittrich. Prager Vierteljahrschrift für praktische Heilkunde, 1849, 6 Jahrgang, Bd. I., p. 157.
Kaulich, Jos. Prager medicinische Wochenschrift, 1884, IX., p. 505.
Leo. Deutsche medicinische Wochenschrift, 1886, XV., p. 253.
Grummach, E. Berliner klinische Wochenschrift, 1890, p. 22.
Wallach, J. Archiv. f. physiologische Heilkunde, 1852. XI. Jahrgang, S. 3.
Weiss. Deutsches Archiv. für klin. Medicine, 1875. XVI., p. 379.
Fereal. L'Union Medicale, 1881, p. 361.
Fraenkel. Bulletin de la Societé atomique de Paris, 1896, p. 306.
Young. Medicine. June, 1897.
Nixon. The Dublin Journal of the Medical Sciences, 1879. Vol. LXVII., p. 417.
Vèlon. Le Progrés Medical, 1885, p. 423.
Ackerman. Deutsche Klinik, 1870, p. 7.
Jakobowitsch. Züricher Dissertation, 1897.
Bozannis. Würzburger Dissertation, 1876.
Brauner. Münchener Dissertation, 1879.
Achilles. Würzburger Dissertation, 1879.
v. Rad. Tubinger. Dissertation, 1893.
Rickards. The British Medical Journal, 1881, p. 916.
Voss. Schmidt's Jahrbücher. Bd. XCVIII., p. 303.
Howship, Farrel-Watson, Raule, Chasmal, Mansfield, Dubnell, Sibbard.
Mannkopf. Annalen des Charité Krankenhauses. 1862.
Wyss. Correspondenzblatt für schweizer Aerzte, 1871, p. 43.
Ashby, H. Med. Times and Gazette, Vol. I., 1884, p. 353.
Montard-Martin. Union Medicale, 1883, p. 825.
Casey. Le Progres Medicale, 1879, p. 263.
O'Sullivan. The Dublin Journal of Medical Science, 1880, p. 350.
Hall. Wiener Med. Jahrb., 1880, p. 453.

Nasaraw. Referat. Centralblatt f. in. Medicin, 1897, p. 14.
Passow. Charité Annalen, 1894, p. 529.
Hadden. Cited from Jadubowitsch.
Orth. Virchow's Archiv., 1880, p. 529.
Schmidt-Kirsch. Bonner Dissertation, 1892.
Aberle.

388 FRANKLIN STREET.

